# Identification of Stem Leydig Cells Derived from Pig Testicular Interstitium

**DOI:** 10.1155/2017/2740272

**Published:** 2017-01-24

**Authors:** Shuai Yu, Pengfei Zhang, Wuzi Dong, Wenxian Zeng, Chuanying Pan

**Affiliations:** College of Animal Science and Technology, Northwest A&F University, Yangling, Shaanxi 712100, China

## Abstract

Stem Leydig cells (SLCs), located in the testicular interstitial compartment in the mammalian testes, are capable of differentiating to testosterone-synthesizing Leydig cells (LCs), thus providing a new strategy for treating testosterone deficiency. However, no previous reports have identified and cultured SLCs derived from the pig. The aim of the current study was to isolate, identify, and culture SLCs from pigs. Haematoxylin and eosin staining and immunochemical analysis showed that SLCs were present and that PDGFR*α* was mainly expressed in the pig testicular interstitium, indicating that PDGFR*α* was a marker for SLCs in the neonatal pig. In addition, reverse transcription-PCR results showed that SLC markers were expressed in primary isolated LCs, indicating that they were putative SLCs. The putative SLCs were subsequently cultured with a testicular fluid of piglets (pTF) medium. Clones formed after 7 days and the cells expressed PDGFR*α*. However, no clones grew in the absence of pTF, but the cells expressed CYP17A1, indicating that pTF could sustain the features of porcine SLCs. To summarize, we isolated porcine SLCs and identified their basic characteristics. Taken together, these results may help lay the foundation for research in the clinical application of porcine SLCs.

## 1. Introduction

Testosterone not only maintains the spermatogenesis process, but also protects the function of androgen-dependent tissues [1, 2]. As the primary source of synthesizing and secreting testosterone in mammalian testes, adult Leydig cells (ALCs) play essential roles in maintaining vital movement [3]. Recently, it has been demonstrated that ALCs arise from stem Leydig cells (SLCs) [4]. SLCs, which are located in the interstitial compartment close to the seminiferous tubules in mammalian testes, are one of the most important somatic stem cells types [5, 6].

SLCs were firstly identified and enriched from neonatal rat testes by Ge et al. (2006), and further studies demonstrated that putative mouse and human SLCs had the capacity to differentiate into testosterone-producing cells [4, 7, 8]. According to these previous studies, some characteristics of SLCs were identified [4, 9, 10]. First, the number of mammalian SLCs was fairly small; for example, an average of only 8,500 putative SLCs were obtained from one postnatal 7-day-old rat testes [4]. Second, the SLCs residing on the outer surface of the seminiferous tubules in rat testes were spindle-shaped in situ [4, 10]. In addition, putative SLCs expressed LIF receptor (LIFR), platelet-derived growth factor receptor *α* (PDGFR*α*), Nestin, Thy-1, and some stem cell markers; however, they were 3*β*-HSD- and luteinizing hormone receptor- (LHR-) negative [4, 8, 10, 11]. Unfortunately, no SLCs studies had been carried out in other mammalian animals except in rats, mice, and humans.

With increasing age, the number of functional LCs decreased, and the ability for testosterone production, cAMP production, and the activities of steroidogenic enzymes is reduced [12, 13]. Thus, male infertility diseases may occur in older males as a result of LCs dysfunction or testosterone disorder [14, 15]. Currently, androgen-replacement was the most efficient therapy for rescuing testosterone deficiency; however, it required successive treatments and carries inherent risks [16]. SLCs had the ability to self-renew and differentiate into LCs, therefore, providing a new strategy for treating these diseases by SLCs transplantation [17].

The pig had played a crucial role as a mammalian model in human disease studies [18, 19]. The pig testis had been suggested as “the most versatile steroid producing organ known” and provided important material to research the physiology and genetics of human steroidogenesis [20]. However, porcine SLCs had yet to be isolated and enriched. Additionally, species distinctions complicated the studies of porcine SLCs, since completely mapping of the markers and culture systems of rat and mouse SLCs to porcine SLCs had not been achieved. Owing to the importance of porcine SLCs in clinical applications, the objective of this study was to isolate, identify, and culture SLCs from neonatal pig testes.

## 2. Materials and Methods

### 2.1. Collection of Porcine Testes

The study was approved by the Animal Care and Use Committee of Northwest A&F University in accordance with the Guide for the Care and Use of Laboratory Animals of the National Institutes of Health, China. Fresh testes samples of 7-day-old male pigs from Besun agricultural industry group Co., Ltd. (Yangling, Shaanxi, China) were transported to the laboratory in Dulbecco's phosphate-buffered saline (DPBS) supplemented with 2% Penicillin-Streptomycin (P/S) solution (Invitrogen, Carlsbad, CA, USA) within 1 h. Testes samples of 2-month-old male pigs were collected from a pig breeding farm in Yangling, Shaanxi Province, China.

### 2.2. Isolation of Porcine SLCs

An enzymatic digestion method was used for obtaining porcine SLCs. Testes were first washed and minced after the epididymis and tunica albuginea were removed. Then the testicular fragments were suspended in 0.75 mg/mL collagenase type IV (Invitrogen) containing 5% (v/v) fetal bovine serum (FBS, Gibco, UK) plus DNase I (100 *μ*g/mL; Bio Basic, Markham, Canada) and incubated, with constant shaking at 34°C for 90 min [21]. The 160 and 59 *μ*m monofilament nylon meshes (Solarbio, Beijing, China) were then used to filter the cell suspension [21]. The isolated cells were treated with 1 mg/mL hyaluronidase (Invitrogen) and centrifugation at 500*g* for 5 min at 20°C. After 5 min stilling, the upper side of the suspensions was cultured in media for another 15 min stilling. The cells on the upper side of the suspensions were then collected. Finally, the isolated LCs were cultured in Dulbecco's modified eagle medium: nutrient mixture F-12 (DMEM/F12, Invitrogen) medium.

### 2.3. Preparation of Testicular Fluid of Piglets (pTF)

The pTF and primary LCs were derived from the same source. The testes of 7-day-old pigs were cut into fragments as small as possible and pTF was extracted by tissue homogenization at 20°C [22]. Finally, the pTF was filtered through a 0.22 *μ*m strainer to degerm.

### 2.4. Culture of Porcine Isolated LCs

The isolated LCs precipitates were resuspended in two media: one basic medium and the other pTF medium (basic medium plus 30% (v/v) pTF) [22]. The basic medium consisted of DMEM/F12, 10% (v/v) FBS, 1% (v/v) P/S, and 1% (v/v) vitamins. The LCs were then incubated in an atmosphere of 95% air-5% CO_2_ at 34°C and cultured for at least 2 weeks. The culture media were changed daily.

### 2.5. Ethane Dimethanesulphonate (EDS) Treatment

The EDS was provided by Professor Yuanqiang Zhang (Department of Human Anatomy, Histology and Embryology, The Fourth Military Medical University, China). According to the previous methods, EDS was dissolved in dimethyl sulfoxide (DMSO)/sterile water (1 : 3, v/v) [23–25]. Afterwards, the primary isolated SLCs were seeded into a 6-well plate and 0, 0.5, 0.75, and 1.0 mg/mL EDS (final concentration) were added to the culture solution, respectively [24, 25]. Quantitative real time-PCR (qRT-PCR) and immunofluorescent analyses were carried out 24 h after EDS treatment.

### 2.6. Haematoxylin and Eosin (H&E) Staining and Immunohistochemistry Analysis

Testis samples of 7 days' and 2 months' old male pigs were fixed, dehydrated, and embedded in paraffin. The paraffin-embedded tissues were then sectioned at 5 *μ*m using standard procedures and adhered to precoated glass slides. Afterwards, H&E staining of the paraffin-embedded sections was conducted to observe the histology [26].

For immunohistochemistry, PDGFR*α* expression in the interstitial cells of 7-day-old porcine testes and the type of these protein-positive cells was determined. In detail, the paraffin sections were deparaffinized, rehydrated, and rinsed in PBS. Then antigen retrieval involved boiling of the samples in a solution of 0.01 M Tris-ethylenediamine tetraacetic acid (Tris-EDTA; pH = 9.0) for 10 min. The sections were incubated with 10% donkey serum for 2 h at 37°C, followed by incubation with primary antibodies (anti-PDGFR*α*, 1 : 200, Abcam, Cambridge, UK) overnight at 4°C and subsequent incubation with secondary biotinylated antibodies (ZSGB-BIO, China) for 1 h at 37°C [27, 28]. Afterwards, 3,3′-diaminobenzidine (DAB, ComWin Biotech, China) was used as a chromogen to detect protein expression.

The characteristics of the isolated cells were detected by immunofluorescence staining. First, cells were fixed with 4% paraformaldehyde and permeabilized with 0.05% Triton X-100 for 15 min. The cells were then incubated with primary antibodies at 4°C overnight, and then for 2 h with appropriate Alexa Fluor 594-conjugated secondary antibodies (1 : 400, Invitrogen, USA) at 37°C. Finally, the cells were labeled with 4,6-diamidino-2-phenylindole (DAPI, 1 : 1000; Beyotime, China). The primary antibodies used were rabbit anti-PDGFR*α* (1 : 200, Abcam) and mouse anti-CYP17A1 (1 : 100, Santa Cruz, USA).

All images of all the staining were captured using a Nikon Eclipse 80i fluorescence microscope camera (Tokyo, Japan).

### 2.7. qRT-PCR Analysis

Total RNA were extracted from cells and porcine testes tissues using RNAiso Plus reagent (TaKaRa, Dalian, China) according to the recommended protocol. The cDNA was then synthesized for reverse transcription PCR (RT-PCR) using the PrimeSript™ RT reagent Kit (TaKaRa). Specific primers (Table 1) were used to characterize the isolated cells. The qRT-PCR reaction system was 20 *μ*L in volume: 10 *μ*L SYBR® Premix Ex* Taq* II (2x) (TaKaRa), 0.8 *μ*L cDNA, 0.5 *μ*L PCR Forward Primer (10 *μ*mol/L), 0.5 *μ*L PCR Reverse Primer (10 *μ*mol/L), and added sterile water to total volume of 20 *μ*L. PCR reaction conditions were as follows: denaturation at 95°C for 3 min, followed by 40 cycles of (95°C for 15 s, 60°C for 30 s, and 72°C for 30 s).

### 2.8. Oil Red O Staining

For the visualization of the lipid droplets, LC was fixed in 4% formaldehyde (freshly prepared from paraformaldehyde) for 15 min, stained in Oil red O staining solution (0.3% Oil Red O solution) for 10 min, and then washed with PBS 2~3 times. The cells were then captured using a Nikon Eclipse 80i fluorescence microscope camera.

### 2.9. Statistical Analyses

The mRNA expressions detected by qRT-PCR were calculated using the 2^−ΔΔCT^ method and normalized by the expression of*β-actin* [29]. The mRNA expression variation between different samples was calculated using SPSS (version 18.0) (SPSS, Inc., Chicago, IL, USA). Statistical differences of genes in different groups were determined by ANOVA, and the data were presented as mean ± standard deviation of duplicates.

## 3. Results

### 3.1. SLCs Were Present in the Neonatal Porcine Testes

**Figure 1:**
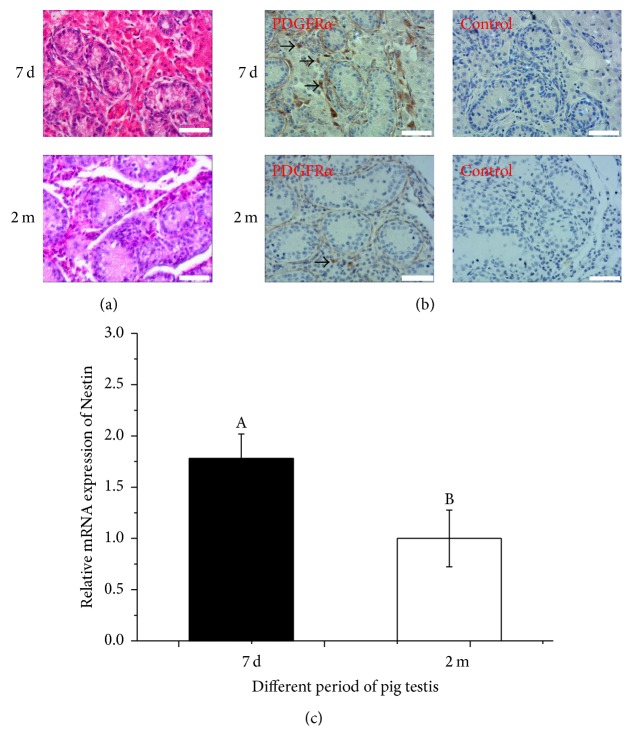
Identification of pig stem Leydig cells (SLCs) in situ. (a) H&E staining of 7 days' and 2 months' old porcine testes (bar = 50 *μ*m). (b) Immunohistochemical analysis of PDGFR*α* of 7 days' and 2 months' old porcine testes (bar = 50 *μ*m); the black arrowheads indicated the PDGFR*α*-positive cells in testicular interstitium. (c) mRNA expression of* Nestin* in 7 days' and 2 months' old pig testes. Different letters (A, B) indicate significant difference (*P* < 0.05).

A number of spindle-shaped cells were found in the testicular interstitium in the postnatal 7 days' and 2 months' old porcine testes by H&E staining (Figure 1(a)). Furthermore, immunochemical analyses showed that PDGFR*α* was mainly expressed in the testicular interstitium in postnatal 7-day-old pigs, while the expression of PDGFR*α* was low in the 2-month-old porcine testicular interstitium (Figure 1(b)). Moreover, the expression of* Nestin* in the 7-day-old porcine testes was significantly higher than that in the 2-month-old testes (*P* < 0.5) (Figure 1(c)). Based on these results, we chose to collect SLCs from 7-day-old pigs rather than 2-month-old pigs.

### 3.2. The Isolated LCs from Porcine Testicular Interstitium Expressed Markers of SLCs

**Figure 2:**
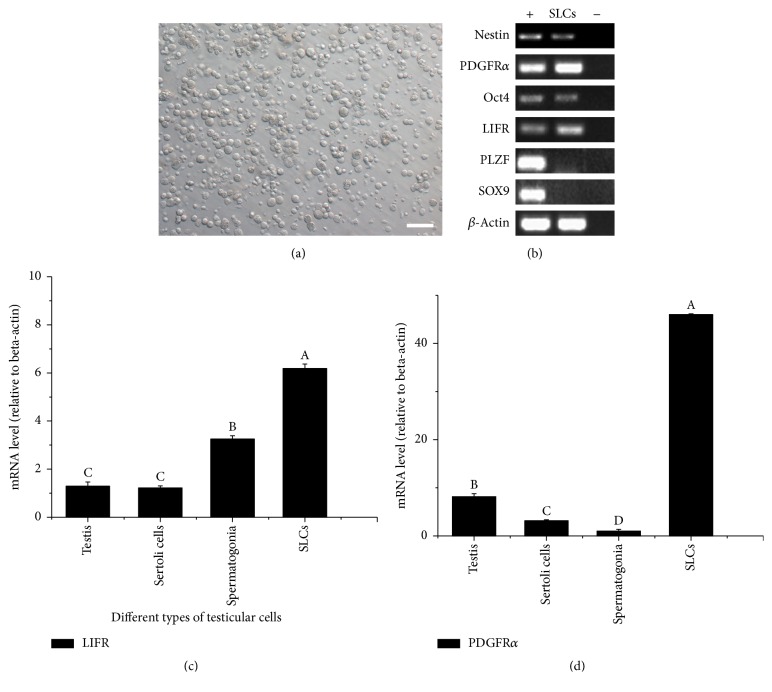
Identification of porcine SLCs. (a) The primary SLCs isolated from 7-day-old porcine testes with the help of collagenase type IV (bar = 50 *μ*m). (b) RT-PCR results of genes involved in stem cells potential and spermatogenesis; SLCs, the pig primary isolated SLCs; +, positive control (7-day-old pig testes); −, negative control (sterile water). (c and d) Expressions of* LIFR* and* PDGFRα* in pig testes, pig Sertoli cells, pig Spermatogonia stem cells and pig primary isolated SLCs as fold change relative to beta-actin; Spermatogonia, Spermatogonia stem cells. Different letters (A, B) indicate significant difference (*P* < 0.05).

The primary LCs were obtained by digestion method (Figure 2(a)). RT-PCR and immunofluorescent analysis were then used to characterize these cells. As shown in Figure 2(b), RT-PCR results showed that the isolated LCs expressed SLCs or pluripotency stem cell markers (Nestin, PDGFR*α*, GATA-4, Oct4, and LIFR) (Figure 2(b)). Moreover, markers of Sertoli cells (SOX9) and spermatogonial stem cells (PLZF) were not detected (Figure 2(b)), indicating no contamination with these cells in these LCs. The results demonstrated that this digestion method was useful in removing the seminiferous tubules. Moreover, qRT-PCR results showed that the expressions of* LIFR* and* PDGFRα* in the LCs were significantly higher than that in the porcine testes (*P* < 0.5) (Figures 2(c) and 2(d)), indicating that this method was able to enrich SLCs from porcine testes. In summary, the primary isolated LCs, expressing SLCs markers (Nestin, PDGFR*α*, GATA-4, Oct4, and LIFR), were putative SLCs.

EDS was used to specifically eliminate differentiated LCs in rat and mouse testes [4, 8]. According to results of the EDS treatment, the percentage of porcine differentiated LCs was approximately 23% in the primary isolated LCs, and the purity of primary isolated porcine SLCs was over 77% (Figures S1, S2, in Supplementary Material available online at https://doi.org/10.1155/2017/2740272). In addition, qRT-PCR results of* Nestin*,* PDGFRα*,* CYP17A1* expressions, and the immunofluorescent analysis of CYP17A1 further confirmed that EDS could specifically eliminate differentiated LCs in the pig (Figures S3, S4), which was consistent with the results of cell survival rates after EDS treatment (Figure S2).

### 3.3. These Isolated SLCs Exhibited High Clonogenic Potential

**Figure 3:**
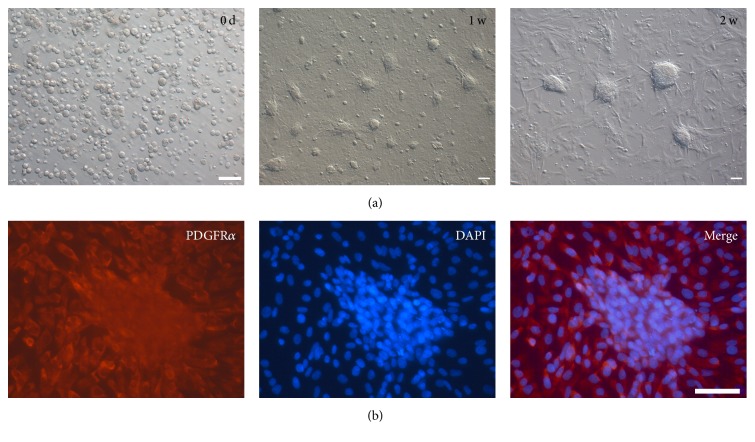
Morphology development and PDGFR*α* immunofluorescence analysis of porcine SLCs cultured in pTF medium (bar = 50 *μ*m). (a) Morphology development of porcine SLCs cultured 0 d, 1 w, and 2 w in pTF medium. (b) PDGFR*α* immunofluorescence of porcine SLCs cultured in pTF medium for 2 w.

Even though the primary SLCs were isolated, their culture system was yet to be determined. In the current study, pTF was used as the main component in the medium. Seven days later, a number of clones were formed, which grew larger following 2 weeks of culture (Figure 3(a)). Immunofluorescent analysis showed that the clones were PDGFR*α* positive (Figure 3(b)). The expressions of both* Nestin* and* LIFR* were higher in porcine SLCs cultured with pTF medium compared to in SLCs without culture (*P* < 0.5) (Figure 5), indicating that pTF was able to sustain the stem cell potential of SLCs.

### 3.4. Isolated SLCs Showed the Capacity of Spontaneous Differentiation into LCs When Cultured In Vitro

**Figure 4:**
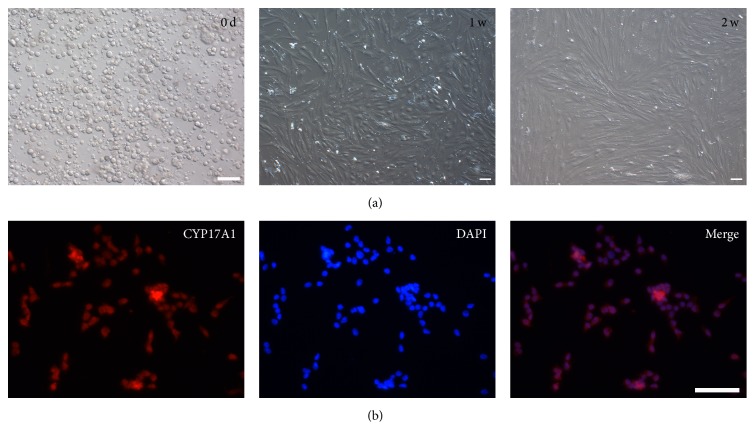
Morphology development and CYP17A1 immunofluorescence of porcine SLCs cultured in basic medium. (a) Morphology development of porcine SLCs cultured 0 d, 1 w, and 2 w in basic medium. (b) CYP17A1 immunofluorescence of porcine SLCs cultured in basic medium for 2 w.

**Figure 5:**
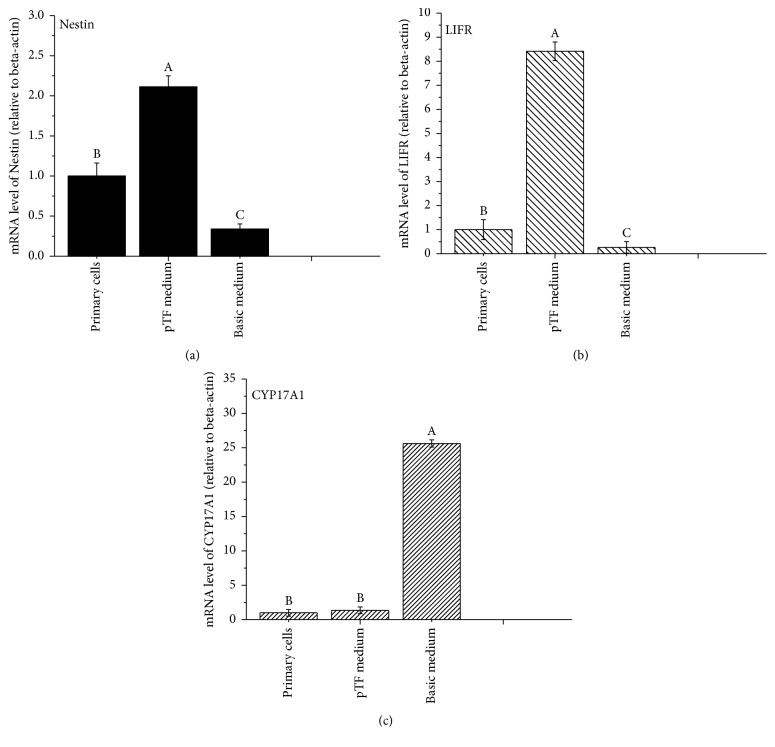
Expressions of* Nestin*,* LIFR,* and* CYP17A1* of porcine SLCs cultured in different media for 2 w. Note: primary cells, the primary isolated porcine SLCs. Different letters (A, B, C) indicate significant difference (*P* < 0.05).

**Figure 6:**
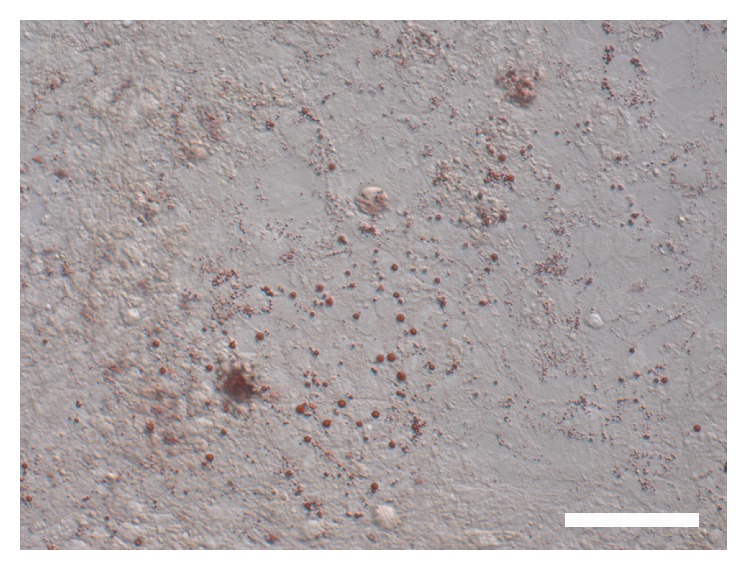
Oil Red O staining of pig LCs after cultured 7 d in the basic medium (bar = 50 *μ*m).

The isolated cells cultured with a basic medium did not form clones after 2 weeks (Figure 4(a)) and expressed CYP17A1, a marker of pig differentiated LCs (Figure 4(b)). Moreover, the expressions of both* Nestin* and* LIFR* were significantly lower in porcine SLCs cultured with the basic medium for 2 weeks compared to in SLCs without culture (*P* < 0.5) (Figure 5). The expression of* CYP17A1* was significantly higher in porcine SLCs cultured with the basic medium for 2 weeks than that of SLCs without culture (*P* < 0.5) (Figure 5). Oil Red O staining showed that the cultured cells secreted lipid droplets, which was also a marker of differentiated LCs (Figure 6). These results demonstrated that the primary isolated SLCs were able to differentiate into LC lineages when cultured with the basic medium, indicating that the putative SLCs had capacity to spontaneously differentiate into LCs.

## 4. Discussion

Several cell types were essential for spermatogenesis in the testis: germ cells, Sertoli cells, peritubular myoid cells, and ALCs [30]. ALCs were the major source of testosterone secretion in mammals; however, they were incapable of proliferation. Testosterone could diffuse into Sertoli cells so that it indirectly regulated spermatogenesis. When the processes of synthesizing testosterone were disturbed, postmeiotic spermatids were significantly reduced or absent [30]. SLCs were therefore ideal for rescuing infertility caused by LCs dysfunction. In addition, it had been demonstrated that SLCs were able to differentiate into ALCs in vivo by transplanting the alginate-encapsulated interstitial tissue into rat extra-testis tissue [31]. Therefore, mammalian SLCs held great promise for research and clinical use in male infertility.

Recently, SLCs had been successfully isolated from rats, mice, and humans, but not from pigs. Previous studies had shown that several proteins were detected in putative SLCs in the rat testicular interstitium, such as Nestin, LIFR, PDGFR*α*, CD90, and CD51 [11, 32]. However, a majority of these were also expressed in other testicular cells, and they made useful markers of SLCs, as they were expressed in a time and/or stage-specific manner. For example, Ge and his colleagues demonstrated that the PDGFR*α*-positive and 3*β*-HSD-negative cells in postnatal 7-day-old rats were putative SLCs [4]. They then concluded that PDGFR*α* was a marker of rat SLCs in the neonatal stage. In this study, SLCs were identified and PDGFR*α* was shown to be expressed in the SLCs using H&E staining and immunochemistry. Moreover, results from immunochemistry and qRT-PCR analysis showed that the expressions of both* PDGFRα* and* Nestin* were significantly higher in postnatal 7 days' than 2 months' old pig (*P* < 0.5). These results predicted that PDGFR*α* could also be used as a marker of neonatal porcine SLCs and the 7-day-old sampling point was more suitable for isolating SLCs than the 2 months old in pigs.

However, no studies had reported the isolation of porcine SLCs. In the rat, Percoll purification and immunoselection technologies were used to obtain SLCs by Ge et al. (2006) [4], and several studies had used transgenic mice to obtain mouse SLCs [8, 9]. In the current study, collagenase and hyaluronidase digestion was used to isolate pig testicular interstitial cells from pig testes. Moreover, hyaluronidase could isolate individual cells from the outer surface of seminiferous tubules. Thus, the method used in the current study was simpler and faster than the methods used in mice and rats.

Like other stem cells, the proliferation and differentiation of SLCs were also regulated by the microenvironment, which provided vital cell factors and proteins. In the testes, some types of cells, such as Sertoli cells and peritubular myoid cells, secreted factors into the testicular fluid to regulate the activities of SLCs [33–35]. Since the culture system of porcine SLCs had not been developed, all factors from whole testes were extracted as pTF. At first, we conjectured that the pTF could maintain the stem cell potential of porcine SLCs when added to the culture medium. The results of this work showed that the pTF could indeed support the stem cell potential of SLCs for 2 weeks in vitro. The pTF was able to maintain the self-renewal properties of SLCs, as the origin of pTF was consistent with the putative SLCs. Moreover, the pTF contained abundant hormones, growth factors, cytokines, and a large amount of proteins, which could provide the necessary material basis for SLCs proliferation [22, 36]. The immunofluorescent analysis of PDGFR*α* also demonstrated that the cells that had been cultured for 2 weeks were putative SLCs. Taken together, the results indicated that the pTF might be contributing to maintaining self-renewal properties of the putative SLCs. Therefore, our future research will be directed towards revealing the vital components for maintaining SLCs self-renewal in pTF.

There were two areas of innovation of the present study. First, it provided a simpler and faster method for obtaining the porcine SLCs, which might provide a reservoir for LCs-lineage differentiation. Second, it developed a new short-term culture system for porcine SLCs. In addition, as an ideal human model, some human drugs toxicity investigations of sterile diseases could be assessed in the pig firstly, before human trials, which could reduce the expense of investigations into new drugs.

## 5. Conclusions

To summarize, we isolated porcine SLCs and identified some of their basic characteristics. Moreover, pTF could maintain the features of porcine SLCs when added to culture system. This work might help us to understand the regulatory mechanisms of proliferation and differentiation of SLCs and holds promise for further studies pertaining to porcine SLCs.

## Supplementary Material

EDS was used to eliminate differentiated LCs in the isolated primary cells in this study. As shown in Figure S1, a part of primary cells dead when incubated with different concentrations of EDS (0, 0.5, 0.75, and 1.0 mg/mL). The dead cell percentage was approximately 23%, which showed that the percentage of differentiated LCs was approximately 23%, and the purity of primary isolated porcine SLCs was over 77% (Figure S1, S2). Then qRT-PCR results showed that the expressions of *Nestin* and *CYP17A1* were statistically significance in 1.0 mg/mL EDS treated group than control group, which predicted that 1.0 mg/mL was the best treatment concentration of EDS in this study (Figure S3). At the foundation of this result, immunofluorescent analysis was used to detected the expression of CYP17A1 in the primary cells before and post EDS treatment. As shown in Figure S4, the percentage of CYP17A1-positive cells was approximately 25% before EDS treatment, which was consistent with the dead cell percentage. Meanwhile, the membrane structures of CYP17A1-positive cells were broke after EDS treatment, predicting that EDS could be eliminate porcine differentiated LCs in this study (Figure S4).

## Figures and Tables

**Table 1:** Primer sequences for genes designed and used in this study.

Primers	Primer sequences (5′-3′)	Length of production/bp	Notes
LIFR	F: TAGCACGTGAATTGCGGACT	117	RT-PCR & qRT-PCR
	R: CAGTGCAACAACGAATGCGA		
Nestin	F: GGAGAAACAGGGCCTACAGAG	112	RT-PCR
	R: TAGGAGGGTCCTGTATGTGGC		
GATA-4	F: AATCGAAGACGTCAGCAGGT	123	RT-PCR
	R: GCTCTGTCTTGATGGGACGC		
Oct4	F: GTGTTCAGCCAAACGACCATC	143	RT-PCR
	R: GTCTCTGCCTTGCATATCTCC		
PDGFR*α*	F: GTGGAGAATCTGCTGCCTGG	133	RT-PCR & qRT-PCR
	R: TGTAGGTGACGCCGATGTAG		
PLZF	F: GCGGAAGACCTGGATGACCT	105	RT-PCR
	R: GTCGTCTGAGGCTTGGATGGT		
SOX9	F: GCAAACTCTGGAGACTGCTGAATG	137	RT-PCR
	R: GCCGTTCTTCACCGACTTTCTC		
CYP17A1	F: ATTGACTCCAGCATTGGCGA	179	RT-PCR & qRT-PCR
	R: CCGAAGGGCAAGTAGCTCAA		
*β*-actin	F: CTCCATCATGAAGTGCGACGT	114	RT-PCR & qRT-PCR
	R: GTGATCTCCTTCTGCATCCTGTC		
