# Supplementary material for: Identification of Stem Leydig Cells Derived from Pig Testicular Interstitium
Source: Stem Cells Int. 2017 Jan 24;2017:2740272. doi: 10.1155/2017/2740272 (PMC5294379; doi:10.1155/2017/2740272)
Supplement: Supplementary file 1 — EDS was used to eliminate differentiated LCs in the isolated primary cells in this study. As shown in Figure S1, a part of primary cells dead when incubated with different concentrations of EDS (0, 0.5, 0.75, and 1.0 mg/mL). The dead cell percentage was approximately 23%, which showed that the percentage of differentiated LCs was approximately 23%, and the purity of primary isolated porcine SLCs was over 77% (Figure S1, S2). Then qRT-PCR results showed that the expressions of Nestin and CYP17A1 were statistically significance in 1.0 mg/mL EDS treated group than control group, which predicted that 1.0 mg/mL was the best treatment concentration of EDS in this study (Figure S3). At the foundation of this result, immunofluorescent analysis was used to detected the expression of CYP17A1 in the primary cells before and post EDS treatment. As shown in Figure S4, the percentage of CYP17A1-positive cells was approximately 25% before EDS treatment, which was consistent with the dead cell percentage. Meanwhile, the membrane structures of CYP17A1-positive cells were broke after EDS treatment, predicting that EDS could be eliminate porcine differentiated LCs in this study (Figure S4). [file 2740272.f1.doc]

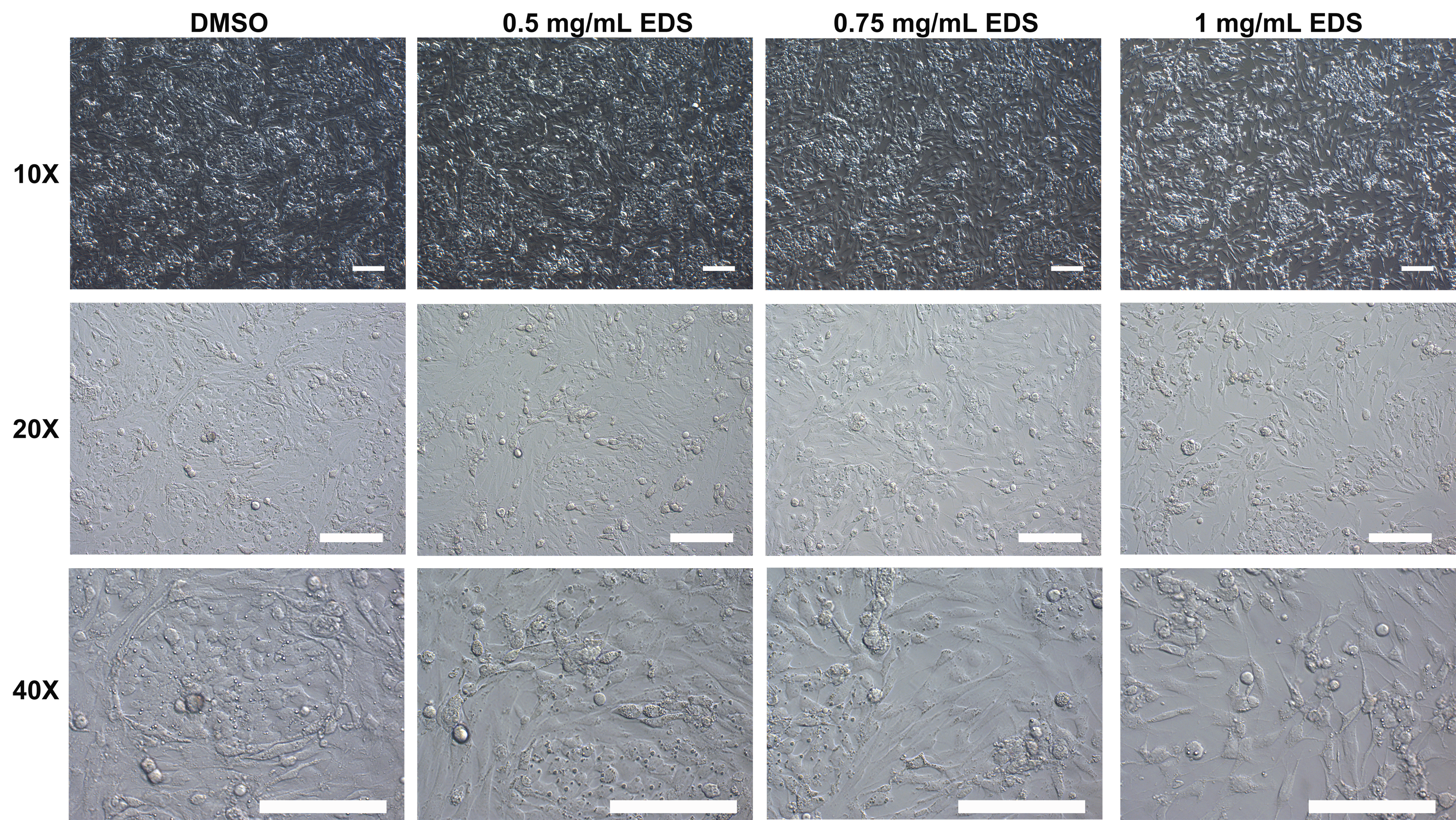


**FIGURE S1 Morphology of porcine SLCs after EDS treatment.** Note: The primary porcine SLCs isolated from 7 days old porcine testes were treated with EDS in different concentrations for 24 h.


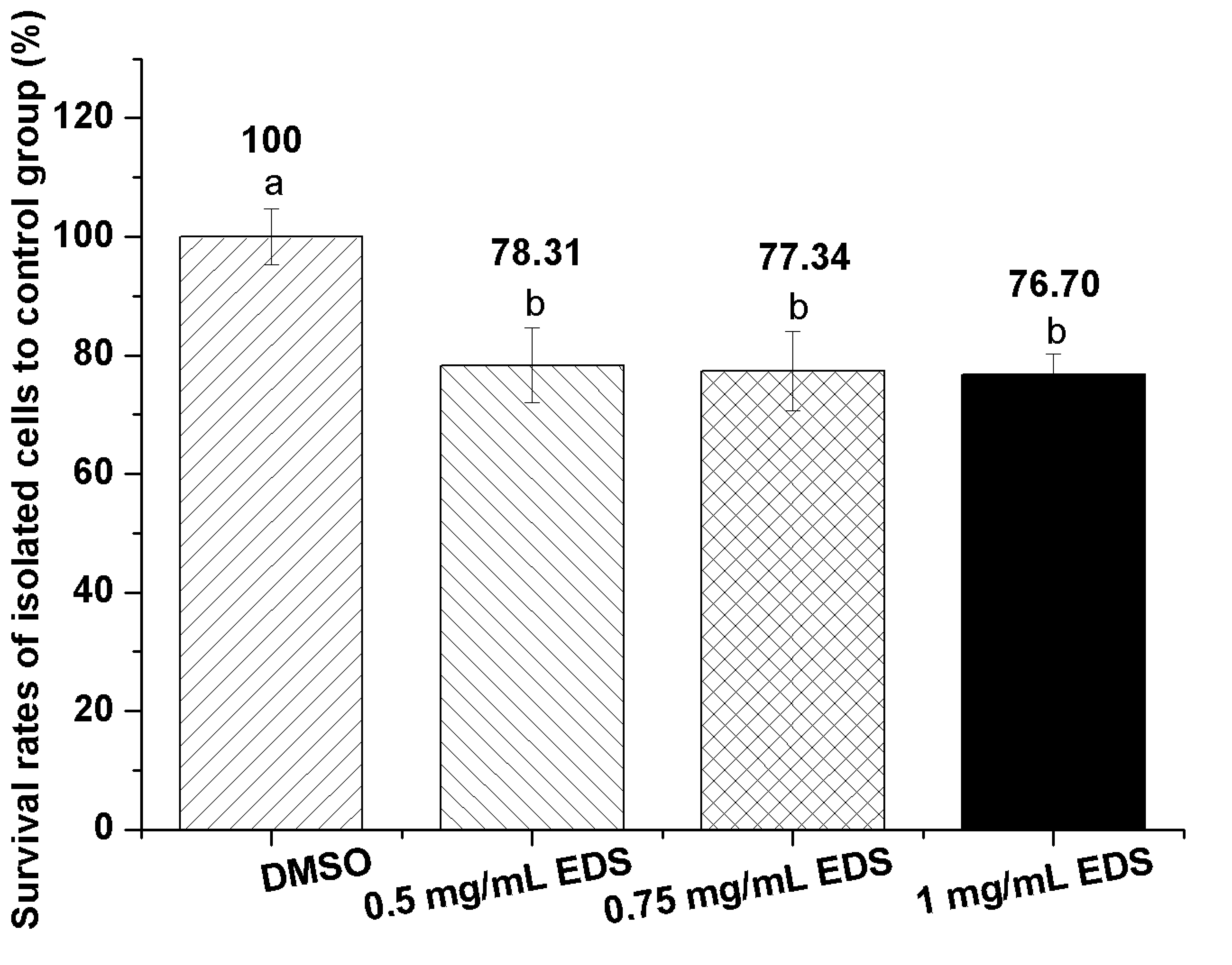


**FIGURE S2 The cell survival rates of SLCs after EDS treatment relative to DMSO treatment.** Note: The primary porcine SLCs isolated from 7 days old porcine testes were treated with EDS in different concentrations for 24 h. Different letters (a, b) indicate significant difference (*P*<0.05).


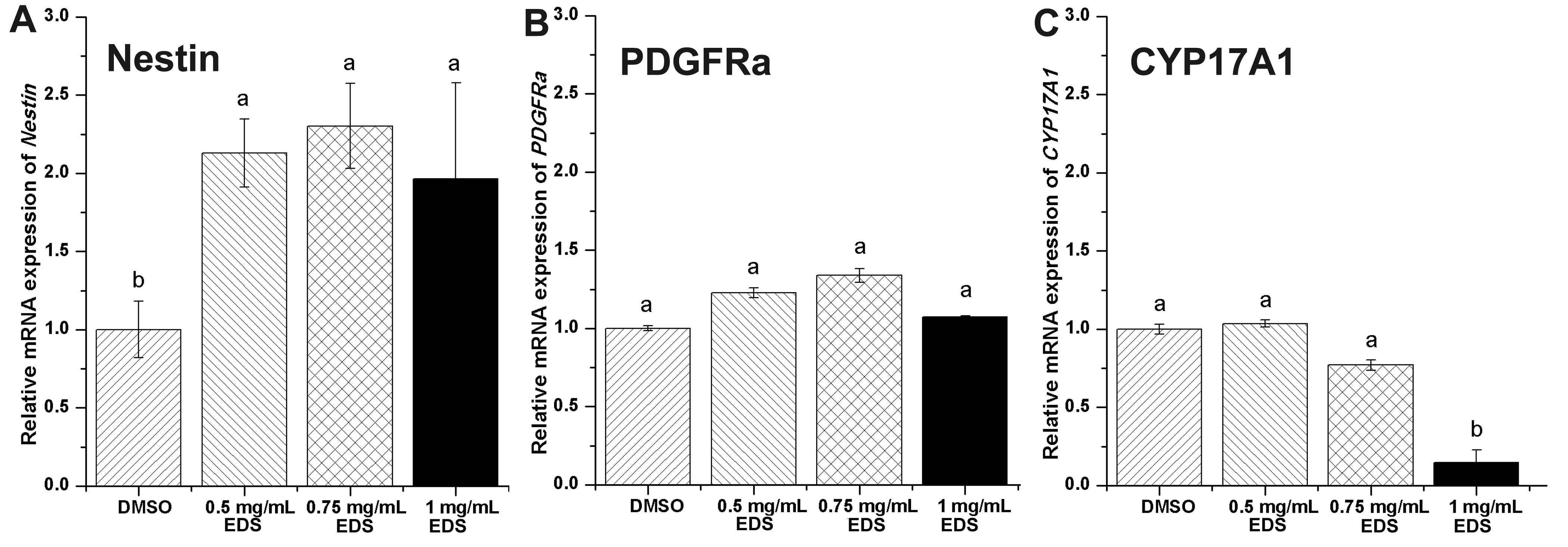


**FIGURE S3 Expressions of *Nestin*, *PDGFRα* and *CYP17A1* of primary porcine SLCs after EDS treatment as fold change relative to DMSO treated group.** Note: The primary porcine SLCs isolated from 7 days old porcine testes were treated with EDS in different concentrations for 24 h. Different letters (a, b) indicate significant difference (*P*<0.05).


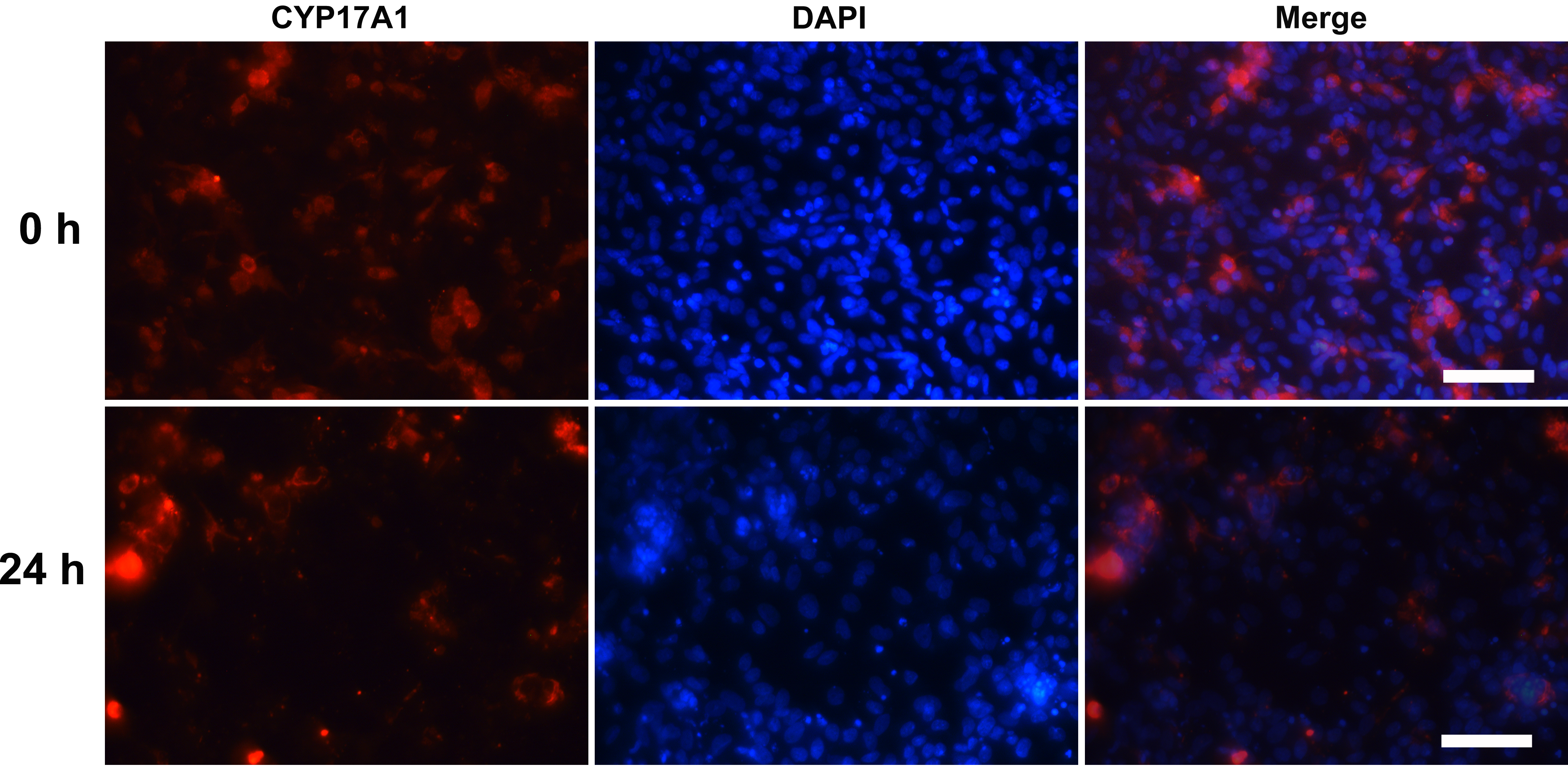


**FIGURE S4 CYP17A1 immunofluorescence of porcine SLCs at 0 h and 24 h of 1.0 mg/mL EDS treatment.** Note: The primary pig LCs were isolated from 7 days old pig testes.
